# A systematic review and meta-analysis of T2, T3 or T4, to evaluate the best denervation level for palmar hyperhidrosis

**DOI:** 10.1038/s41598-017-00169-w

**Published:** 2017-03-09

**Authors:** Wenxiong Zhang, Dongliang Yu, Yiping Wei, Jianjun Xu, Xiaoqiang Zhang

**Affiliations:** Department of Cardiothoracic surgery, The second affiliated hospital of Nanchang University, Nanchang, China

## Abstract

We systematically reviewed and compared the clinical outcomes of thoracoscopic sympathectomy (TS) at different denervation levels for palmar hyperhidrosis. We searched PubMed, Ovid MEDLINE, EMBASE, Web of Science, ScienceDirect, the Cochrane Library, Scopus and Google Scholar for relevant studies published during 1990–2016. Symptom resolution, patient satisfaction, compensatory sweating (CS), recurrence, dry hands and gustatory sweating were assessed. We selected 13 studies from 2228 for the final analysis. A comparison of T2 vs. T3 TS revealed that T3 TS reduced the risk of CS (95% confidence interval [CI]: 1.36–3.19, *p* = 0.0007) and moderate-to-severe CS (95% CI: 2.14–7.87, *p* < 0.0001). No significant differences were found in patient satisfaction, symptom resolution, and incidence of dry hands and gustatory sweating. A comparison of T3 vs. T4 TS revealed that T4 TS reduced the risk of CS (95% CI: 2.87–9.53, *p* < 0.00001), moderate-to-severe CS (95% CI: 2.54–5.83, *p* < 0.00001), dry hands (95% CI: 4.07–18.13, *p* < 0.00001) and gustatory sweating (95% CI: 1.53–7.32, *p* < 0.003), and improved patient satisfaction. No significant differences were found in symptom resolution and recurrence. T4 TS appears to be more useful than T3 or T2 TS for PH.

## Introduction

Palmar hyperhidrosis (PH) is a condition marked by excessive perspiration of hands beyond physiologic need, and is aggravated during periods of stress and anxiety. Thoracoscopic sympathectomy (TS) has become the standard surgical treatment for palmar hyperhidrosis (PH) owing to its association with minimal trauma and low morbidity rates^1, 2^. Conventional surgery to transect the sympathetic chain at the T2–T3 or T2–T4 levels can improve the symptom of PH perfectly well. However, this type of surgery is associated with high incidence rates of complications such as compensatory sweating (CS) and dry hands^3–5^. Various methods have been tried to decrease the incidence of complications after TS, and limitation of the extent of surgical dissection has been found to be the key to achieving this goal. Several authors have performed TS at a single level and achieved a better curative effect (similar resolution of symptoms and lower incidence of CS) than that achieved with TS at multiple levels^6–8^. With the deepening of our understanding of TS, single-level surgeries, especially at the T3 or T4 level, became increasingly common. Although TS at the T4 level has been recommended for the treatment of PH under the Lin-Telaranta classification, the optimal level for TS has been the subject of intense debate among thoracic surgeons, possibly due to a lack of large-scale clinical research in this area^9^. In an attempt to resolve this issue and optimize surgical procedures, we performed a systematic review and meta-analysis of clinical trials investigating TS at the T2, T3 or T4 level for the treatment of PH.

## Materials and Methods

### Search strategy

On July 10, 2016, we conducted an extensive literature search to identify all relevant studies published from January 1990 to July 2016 according to The Preferred Reporting Items for Systematic Reviews and Meta-Analyses. The following databases were scanned: PubMed, Ovid MEDLINE, EMBASE, Web of Science, ScienceDirect, the Cochrane Library, Scopus and Google Scholar. The MeSH terms used included “hyperhidrosis” and “sympathectomy or sympathicotomy”. We also searched the reference lists of the included articles to identify additional studies.

### Inclusion and exclusion criteria

The inclusion criteria were as follows: (1) studies published in English, (2) studies involving patients with either isolated PH or PH combined with hyperhidrosis of other regions, (3) studies comparing the clinical outcomes of TS at different single levels (T2 vs. T3 or T3 vs. T4), (4) studies including ≥10 patients in each group and (5) the most recent study in the case of duplication of data in more than one article.

Reviews without original data, case reports, meta-analyses, letters, expert opinions and animal studies were excluded.

### Data extraction

Data extraction was accomplished by two observers independently, using a standardized Excel form. Any disagreement was resolved the help of a third investigator. The recorded data included the following: first author, year of publication, study design, denervation level, number of patients per group, resolution of symptoms, number of satisfied patients, rate of postoperative complications (recurrence, dry hands and gustatory sweating) and CS (incidence and severity).

### Quality assessment of included studies

Methodological quality was assessed using the Newcastle-Ottawa Scale (NOS) for non-randomized studies and the Jadad scale for randomized controlled trials (RCTs). The NOS (9 points) contained questions for three main items: selection, comparability and exposure. Studies that scored 8–9 points were considered to be of high quality, while those that scored 6–7 points were considered to be of medium quality^10^. The Jadad scale (5 points) evaluates the quality of studies by analysing three items: randomization, masking and accountability of all patients (withdrawals and dropouts). Studies that scored ≥3 points were considered to be of high quality^11^.

### Statistical analysis

We designed two comparisons (T2 vs. T3 and T3 vs. T4) to identify the optimal level of TS. Meta-analysis was conducted using Review Manager 5.3 (The Nordic Cochrane Centre, The Cochrane Collaboration, Copenhagen, Denmark) and STATA 12.0 (StataCorp. LP, College Station, Texas, USA). A *p*-value < 0.05 suggested statistical significance. Between-group differences were compared using analysis of variance in the case of continuous variables or pooled relative risk with 95% confidence interval (CI) in the case of categorical variables. We used the *I* ^*2*^ and Cochran Q statistics to evaluate the between-study heterogeneity. A random-effects model was adopted when the heterogeneity was significant (*p* ≤ 0.10 and *I* ^*2*^ > 50%); otherwise, a fixed-effects model was used. The Egger test based on CS data was used to assess potential publication bias.

## Results

### Search results and quality assessment

**Figure 1:**
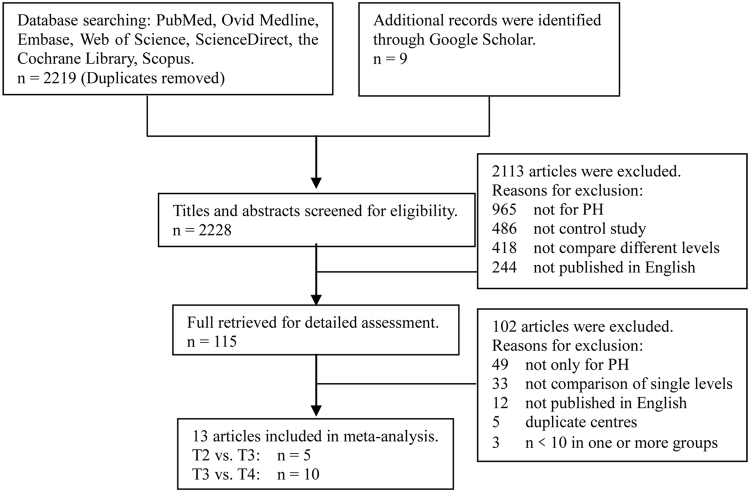
Flow diagram of screened and included papers.

We initially identified 2228 publications from the database and reference-list searches. From these, 13 studies with 1577 patients (261 in the T2 group, 723 in the T3 group and 593 in the T4 group) were selected for final analysis (Fig. 1). Among the 13 studies, 8 were retrospective studies and 5 were RCTs. Quality assessments using the NOS and Jadad scales showed that 10 studies were of good quality, and the other 3 were of medium quality. The baseline characteristics of the included studies and the main evaluation indexes are shown in Table 1.

**Table 1:** Summary of the 13 studies included in the present meta-analysis.

	Study	Levels	NO. of Patients (n)	Resolution (n)	Satisfied cases (n)	CH (n)	Moderate-severe CH (n)	Over dry hands (n)	Gustatory sweating (n)	Recurrence (n)	Design	Quality (score)
2004	Lee^13^	T2	64	60	50	43	26				Retrospective	8
		T3	83	68	57	36	9					
2007	Yang^14^	T3	78	78		55	18			0	RCT	3
		T4	85	85		38	6			0		
2007	Chang^15^	T2	86		78	80		31	5	22	Retrospective	7
		T3	78		75	72		31	18	6		
		T4	70		70	56		6	8	16		
2008	Wolosker^16^	T3	35	34		35	24				Retrospective	8
		T4	35	33		25	3					
2008	Mahdy^17^	T2	20		12	12		7	6	2	Retrospective	9
		T3	20		15	9		4	5	1		
		T4	20		20	2		0	1	0		
2009	Liu^18^	T3	68	68	68	48	9	8			RCT	3
		T4	73	73	73	39	2	1				
2009	Yazbek^19^	T2	30	30		30	10				RCT	4
		T3	30	29		29	3					
2010	Kim^20^	T3	56	56	55	46	6	3	5	1	Retrospective	7
		T4	63	63	61	11	2	0	0	2		
2011	Ishy^21^	T3	20	20		20	1				RCT	4
		T4	20	20		15	1					
2011	Baumgartner^22^	T2	61	58	61	40	4			3	RCT	4
		T3	60	57	59	29	3			3		
2012	Kavakli^23^	T3	43			8				1	Retrospective	6
		T4	46			0				2		
2014	Ellatif^24^	T3	129	129	128	96	28	11		1	Retrospective	8
		T4	145	145	143	41	17	1		2		
2016	Joo^25^	T3	23		17	17	11			8	Retrospective	8
		T4	36		35	22	5			9		

### Main analysis

We comprehensively evaluated the curative effects of TS in two aspects: (1) resolution of symptoms and patient satisfaction and (2) occurrence of postoperative complications (CS, moderate-to-severe CS, recurrence, dry hands and gustatory sweating).

### T2 versus T3

**Figure 2:**
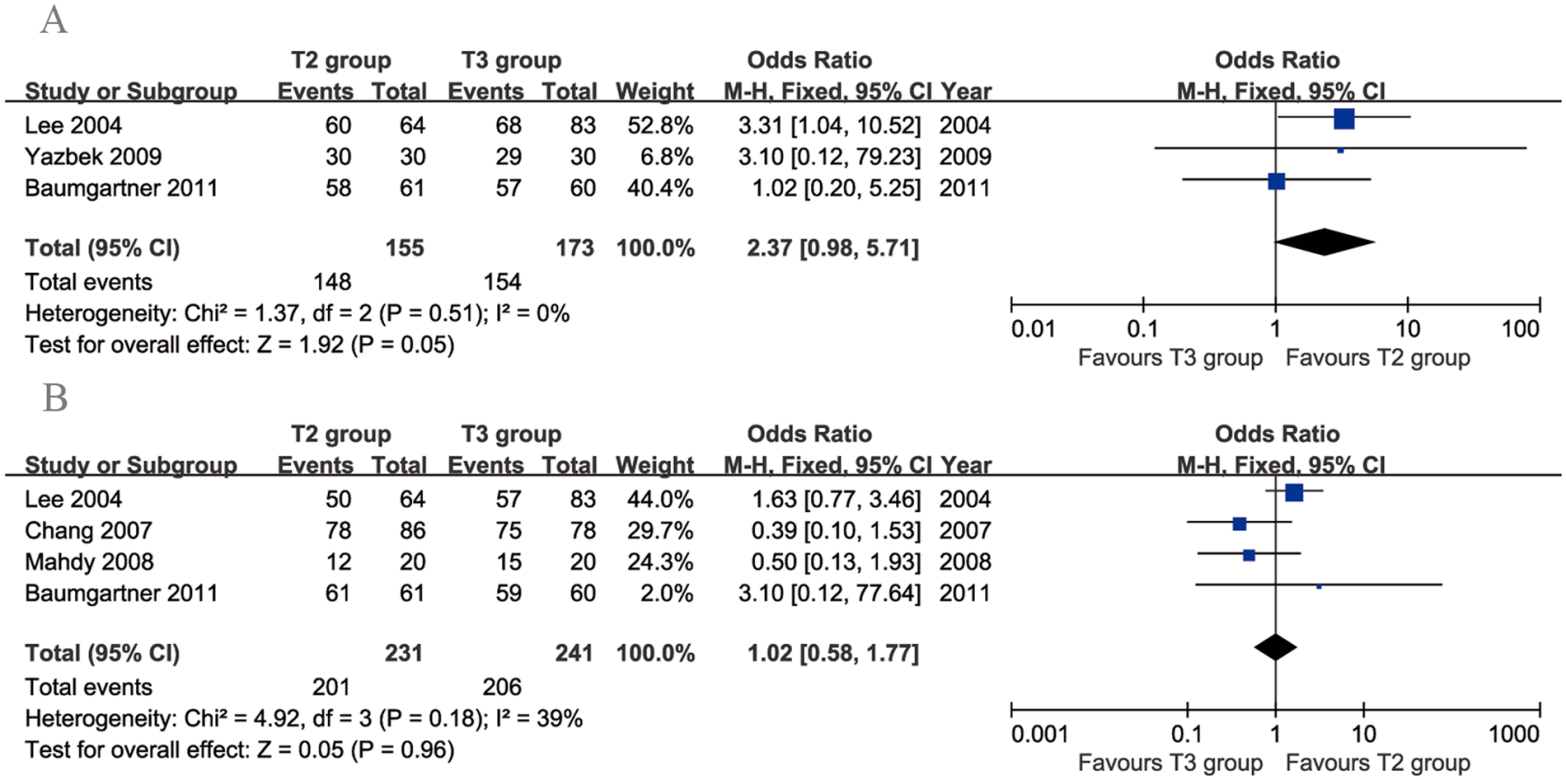
Forest plots for resolution of symptoms (**A**) and patient satisfaction (**B**) in the T2 and T3 groups.

We identified 5 articles that compared T2 vs. T3 TS. They included 261 patients in the T2 group and 271 patients in the T3 group. Data on the resolution of symptoms were available in 3 articles. There was no evidence of heterogeneity among these studies (*p* = 0.51, *I* ^2^ = 0%). No significant difference was found in the resolution of symptoms between the two groups (95% CI: 0.98 to 5.71, *p* = 0.05; Fig. 2A). Four articles compared patient satisfaction between the two groups. The heterogeneity among these studies was acceptable (*p* = 0.18, *I* ^2^ = 39%). No significant difference in the patient satisfaction rate was found between the two groups (95% CI: 0.58 to 1.77, *p* = 0.96; Fig. 2B).

**Figure 3:**
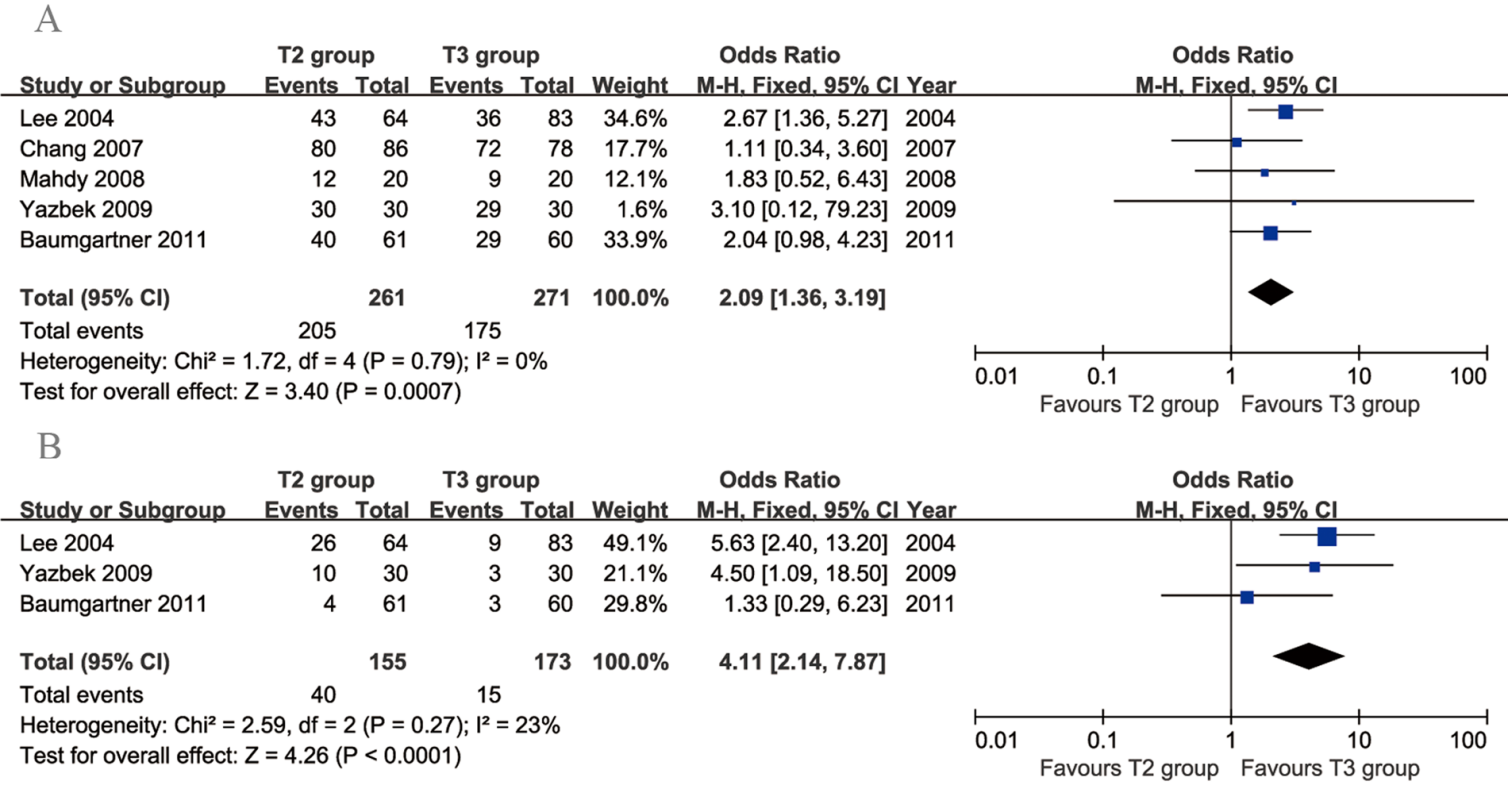
Forest plots for compensatory sweating (**A**) and moderate-to-severe compensatory sweating (**B**) in the T2 and T3 groups.

Five articles compared the rate of CS. There was no evidence of heterogeneity among these studies (*p* = 0.79, *I* ^2^ = 0%). The incidence of CS was significantly lower in the T3 group than in the T2 group (95% CI: 1.36 to 3.19, *p* = 0.0007; Fig. 3A). Three articles compared the rate of moderate-to-severe CS. The heterogeneity among these studies was acceptable (*p* = 0.27, *I* ^2^ = 23%). The incidence of moderate-to-severe CS was significantly lower in the T3 group than in the T2 group (95% CI: 2.14 to 7.87, *p* < 0.0001; Fig. 3B).

**Figure 4:**
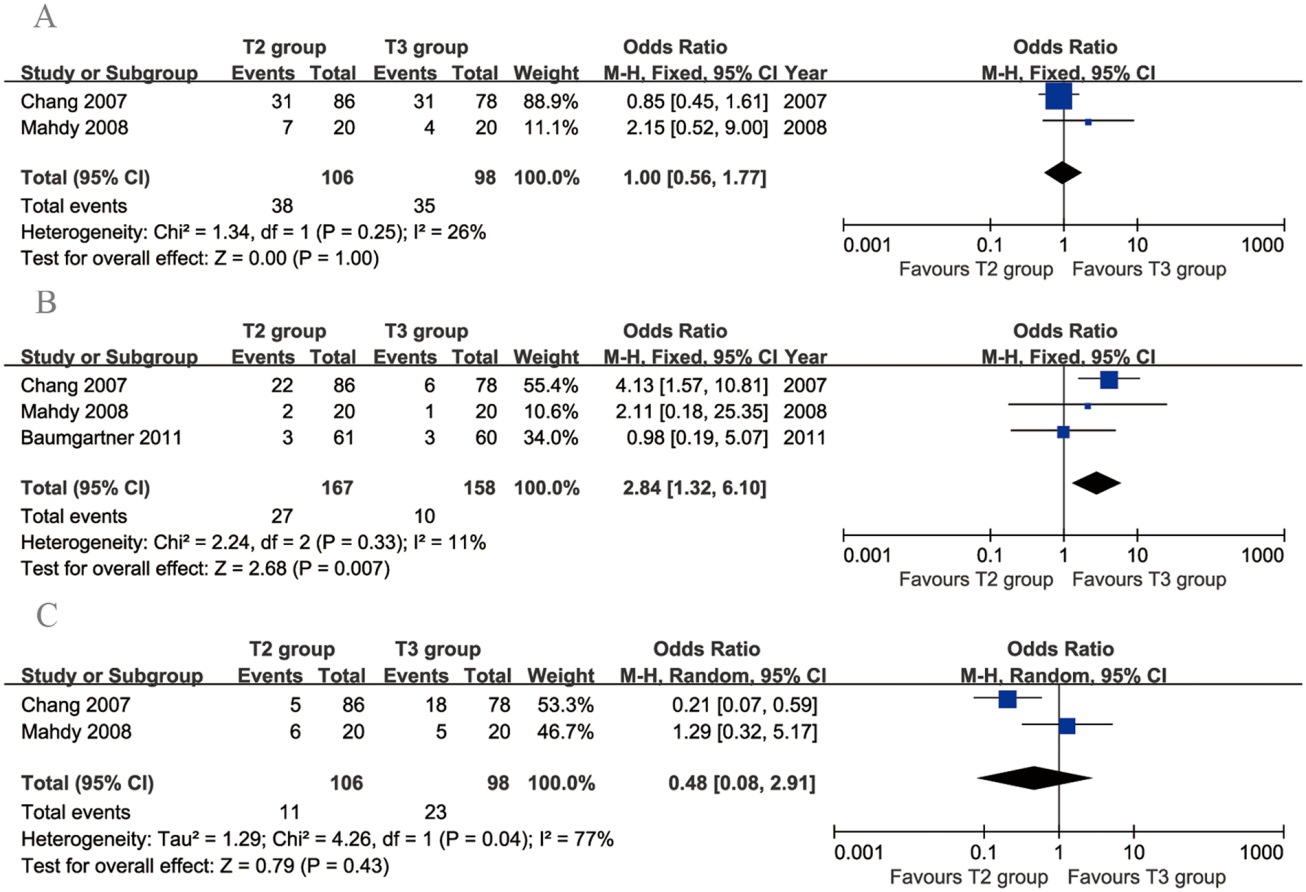
Forest plots for other postoperative complications in the T2 and T3 groups. (**A**) Recurrence, (**B**) dry hands and (**C**) gustatory sweating.

Two articles compared the rate of dry hands. The heterogeneity between these studies was acceptable (*p* = 0.25, *I* ^2^ = 26%). No significant difference was found in the rate of dry hands between the two groups (95% CI: 0.56 to 1.77, *p* = 1.00; Fig. 4A). Three articles compared recurrence rates between T2 and T3 TS. The heterogeneity among these studies was acceptable (*p* = 0.33, *I* ^2^ = 11%). Significantly more recurrences were found in the T2 group than in the T3 group (95% CI: 1.32 to 6.10, *p* = 0.007; Fig. 4B). Two articles compared the rate of gustatory sweating. No significant difference in gustatory sweating was found between the two groups (95% CI: 0.08 to 2.91, *p* = 0.43), but there was significant heterogeneity across the studies (*p* = 0.04, *I* ^2^ = 77%; Fig. 4C).

### T3 versus T4

**Figure 5:**
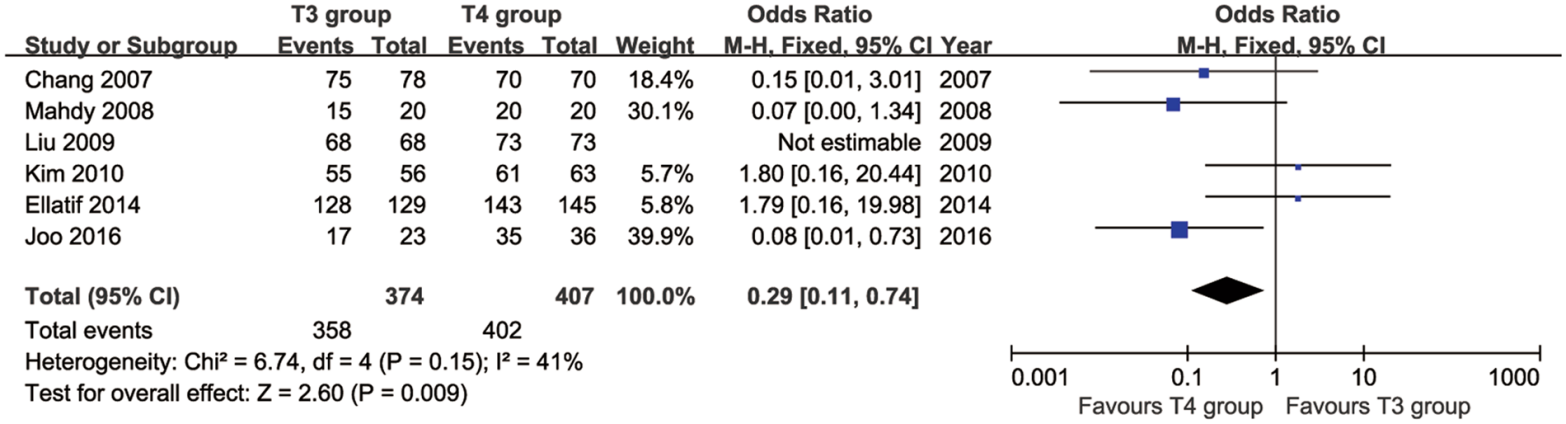
Forest plot for patient satisfaction in the T3 and T4 groups.

We identified 10 articles that compared T3 vs. T4 TS. These studies involved a total of 550 patients in the T3 group and 593 patients in the T4 group. The resolution of symptoms was investigated in 6 studies. Of these, five studies reported that the symptom of PH was resolved in all patients in both groups. Only one study reported that there was no significant difference between the two groups (34/35 vs. 33/35, *p* = 0.56). Satisfaction rates were compared in six papers. The heterogeneity between these studies was acceptable (*p* = 0.15, *I* ^2^ = 41%). Significantly higher satisfaction rates were found in the T4 group than in the T3 group (95% CI: 0.11 to 0.74, *p* = 0.009; Fig. 5).

**Figure 6:**
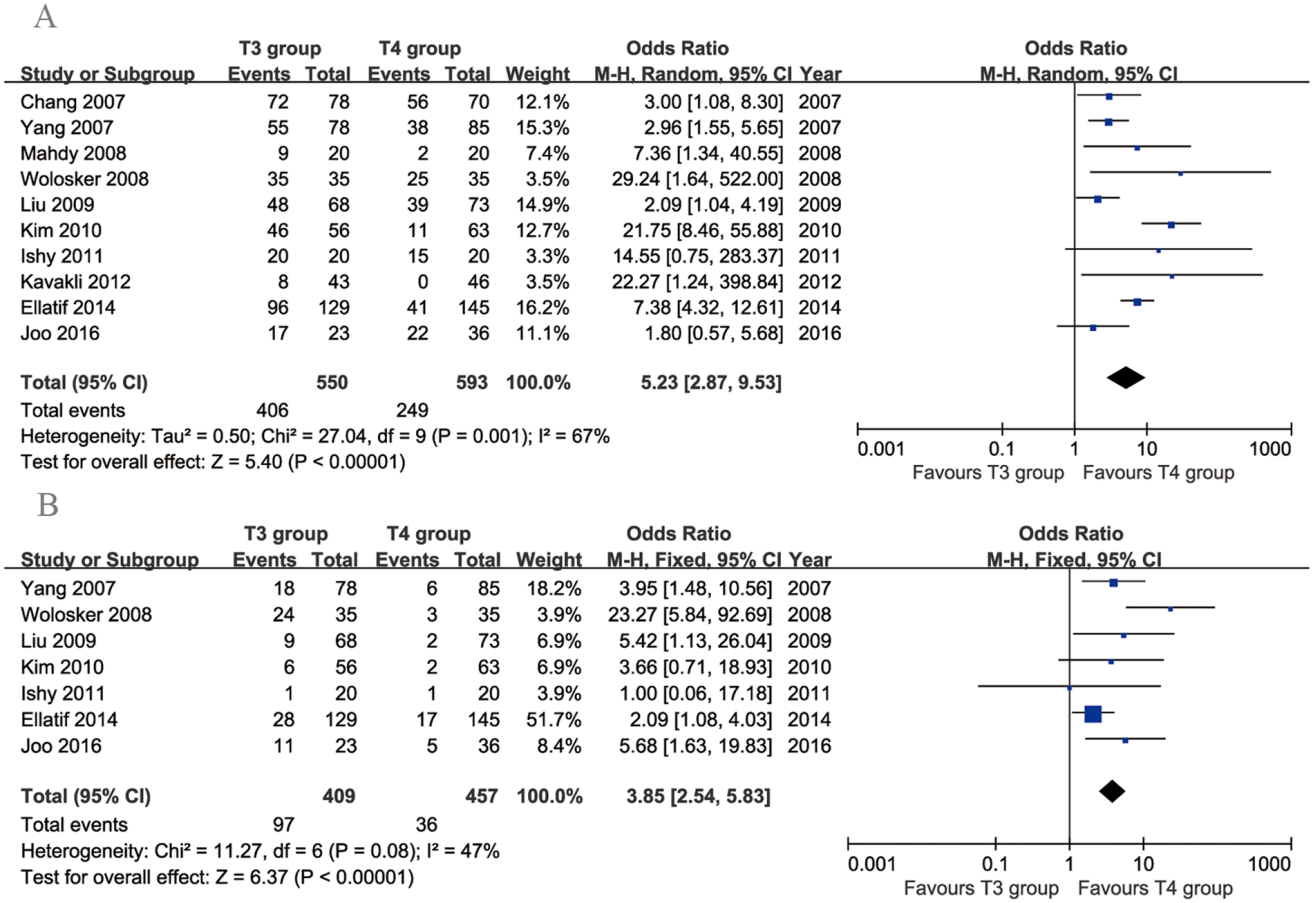
Forest plots for compensatory sweating (**A**) and moderate-to-severe compensatory sweating (**B**) in the T3 and T4 groups.

Ten articles compared CS rates. The incidence of CS was significantly lower in the T4 group than in the T3 group (95% CI: 2.87 to 9.53, *p* < 0.00001), with significant heterogeneity across the studies (*p* = 0.001, *I* ^2^ = 67%; Fig. 6A). Seven articles compared the rates of moderate-to-severe CS. This rate was significantly lower in the T4 group than in the T3 group (95% CI: 2.54 to 5.83, *p* < 0.00001), with acceptable heterogeneity across the studies (*p* = 0.08, *I* ^2^ = 47%; Fig. 6B).

**Figure 7:**
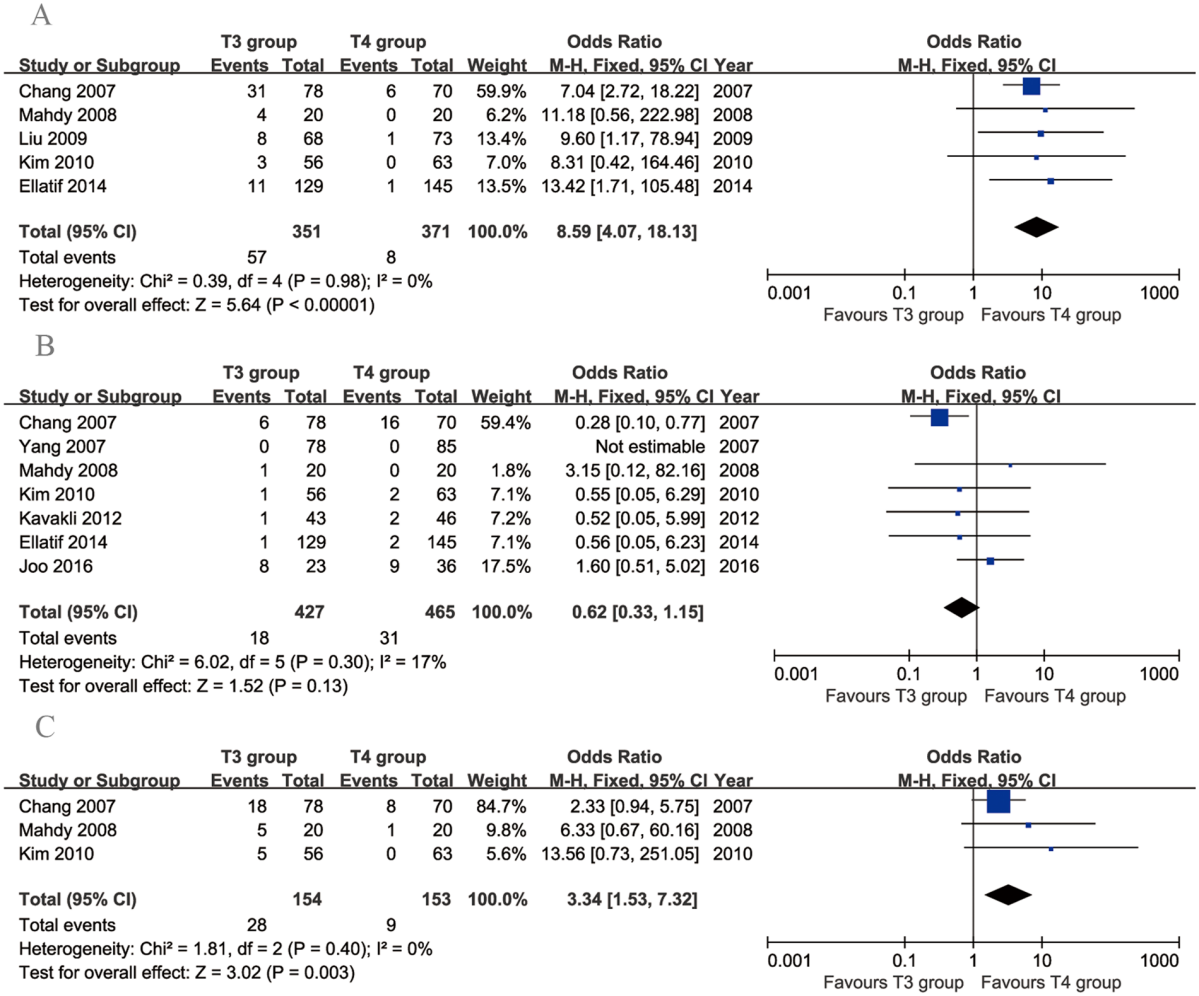
Forest plots for other postoperative complications in the T3 and T4 groups. (**A**) Recurrence, (**B**) dry hands and (**C**) gustatory sweating.

Five articles compared the rates of dry hands. There was no evidence of heterogeneity among these studies (*p* = 0.98, *I* ^2^ = 0%). The incidence of dry hands was significantly higher in the T3 group than in the T4 group (95% CI: 4.07 to 18.13, *p* < 0.00001; Fig. 7A). Seven articles compared recurrence rates between T3 and T4 TS. The heterogeneity among these studies was acceptable (*p* = 0.30, *I* ^2^ = 17%). No significant difference in recurrence rates was found between the two groups (95% CI: 0.33 to 1.15, *p* = 0.13; Fig. 7B). Three articles investigated the incidence of gustatory sweating. There was no evidence of heterogeneity among these studies (*p* = 0.40, *I* ^2^ = 0%). The incidence of gustatory sweating was higher in the T3 group than in the T4 group (95% CI: 1.53 to 7.32, *p* < 0.003; Fig. 7C).

### Publication bias

**Figure 8:**
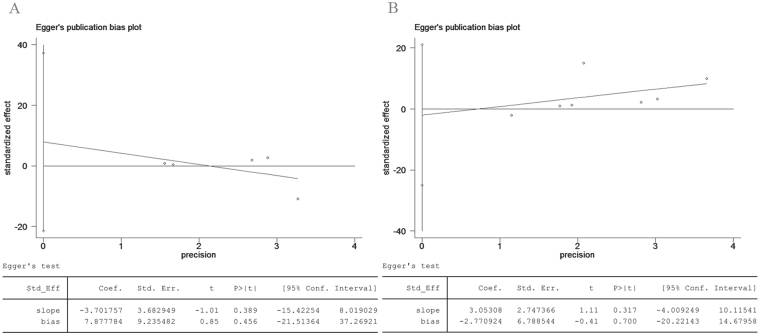
Egger test for compensatory sweating, suggesting no publication bias in the pooled analysis. (**A**) T2 vs. T3 group and (**B**) T3 vs. T4 group.

This study involved two comparisons (T2 vs. T3 and T3 vs. T4), whose publication biases were assessed using the Egger test based on the data for CS. The results showed that there was no significant publication bias (T2 vs. T3, *p* = 0.456; T3 vs. T4, *p* = 0.700; Fig. 8).

## Discussion

TS has been used for the treatment of PH for over 20 years^12^. With our deepening understanding of the disease itself and of the complications associated with TS, the aim of treatment has changed from simply resolving the symptoms to maximizing the quality of life after surgery. Traditional TS at the T2–T3 or T2–T4 levels can improve the symptoms of PH well but also increases the risk of postoperative complications (such as CS and dry hands)^3–5^. Limitation of the denervation level is the most commonly used method to decrease the incidence of complications and achieve satisfactory results^6–8^. In recent years, TS at a single level rather than at multiple levels is increasingly preferred. T2, T3 and T4 are all frequently used denervation levels for PH, and result in fairly good results and a varying incidence of postoperative complications^11, 13–24^. At present, there is still some debate about the optimal transection level for PH. The present meta-analysis therefore assessed 13 studies (5 comparing T2 vs. T3 and 10 comparing T3 vs. T4) in order to provide the most comprehensive evidence for level selection for TS.

Resolution of symptoms and recurrence are the two most important outcomes determining patient satisfaction. Our meta-analysis showed that there was no significant difference in the resolution of symptoms in both comparisons. All three TS levels could achieve a high rate of symptom remission (T2, 95.48%; T3, 96.42%; T4, 99.52%). In the case of recurrence rates, we surprisingly found that T3 was better than T2 and similar to T4. However, only one of the included studies supported this result, and explained that the short duration of follow-up in the T3 group may account for the low recurrence rates in this group^15^. We therefore repeated our analysis after excluding this article, and found no significant differences in both comparisons. Patient satisfaction is the most important criterion to evaluate the success of an operation. Our results suggested that there was no significant difference in patient satisfaction between T2 and T3 TS, and that the satisfaction rate was significantly higher after T4 TS than after T3 TS. The main reasons for dissatisfaction were severe CS and dry hands in the T2 and T3 groups and recurrence in the T4 group^17, 18, 23, 25^.

CS is the most common complication of TS and severely diminishes the postoperative quality of life of the patient^2, 26^. The incidence and severity of CS were regarded as the most important indicators of operative success in most correlational studies. In our study, both the incidence and severity of CS decreased with the descent of the denervation level. The overall incidence of CS was 78.54% in the T2 group, 69.15% in the T3 group and 41.99% in the T4 group, while the incidence of moderate-to-severe CS was 25.81%, 19.24% and 7.88% in the T2, T3 and T4 groups, respectively. The occurrence of CS was believed to be the result of a disturbance in the sympathetic system after the surgery, and greater denervation of the sympathetic nerve might be associated with a higher risk of CS. The preganglionic fibres that innervate the sweat glands of the hands originate mostly from the 2^nd^−6^th^ spinal segments, of which, those from the 3^rd^ and 4^th^ segments are considered to be mainly responsible for PH^27^. TS at the T2 level inevitably cuts off the nerve conduction from the T3 and T4 segments. By the same token, TS at the T3 level cuts off the nerve conduction from the T4 segment. From our results, we consider that the aim of TS should be to transect a certain percentage of sympathetic nerve fibres, but not all. TS at the T4 level might be closer to achieving this aim than TS at the T2 or T3 levels.

Dry hands is a common complication of TS, and if severe, this complication can make patients feel even worse than they did before the operation^15^. In severe cases, the skin on the hands develops cracks, and regular application of hand cream is required to keep the hands moist. In the present meta-analysis, we found that the incidence of dry hands did not differ between the T2 (35.85%) and T3 (16.24%) groups, but was significantly lower in the T4 group (2.16%) than in the T3 group. These results suggest that the incidence of dry hands might be associated with the excessive denervation of sweat glands.

Gustatory sweating has been rarely reported in previous papers. Our results showed that with incidence rates of 10.38% (T2 group), 18.18% (T3 group) and 5.88% (T4 group), gustatory sweating was not a rare side effect. In the study by Herbst, the incidence of gustatory sweating was as high as 50.7%, and was particularly related to spicy or acidic foods^28, 29^. The pathogenesis of gustatory sweating is still unknown. Hashmonai *et al*. have reported that it could result from the sprouting of vagal nerve fibres into the severed sympathetic chain^30^. Our meta-analysis showed that the incidence of gustatory sweating did not significantly differ between the T2 and T3 groups, and was lower in the T4 group than in the T3 group.

Collectively, the above results show that the incidence of postoperative complications (CS, dry hands and gustatory sweating) decreased with the descent of the denervation level. TS at the T4 level could achieve the best curative effect with the lowest incidence of postoperative complications and equally high cure and satisfaction rates. Most of the included studies supported this result and reached a similar conclusion: the lower, the better.

The limitations of our study are as follows: (1) only English articles were included, which might have resulted in a language bias; (2) the sample size of 1577 participants from 13 studies with only 5 RCTs might have weakened the quality of the results; and (3) the operative technique, CS assessment and CS investigation time were similar but not the same across the included studies. These differences might have affected the comparability of the data.

## Conclusion

In conclusion, our analysis suggests that the T2, T3 and T4 sympathectomies were all safe and effective for PH. TS at the T4 level achieved the best curative effect with the lowest incidence of postoperative complications (CS, dry hands and gustatory sweating). However, due to inter-study heterogeneity and the inherent limitations of our meta-analysis, this conclusion requires further validation through more high-quality and large-scale RCTs.
